# Mechanisms of action of Fu Fang Gang Liu liquid in treating condyloma acuminatum by network pharmacology and experimental validation

**DOI:** 10.1186/s12906-023-03960-7

**Published:** 2023-04-20

**Authors:** Zhu Fan, Shuxin Wang, Chenchen Xu, Jiao Yang, Bingnan Cui

**Affiliations:** 1 Guang’anmen Hospital, China Academy of Chinese Medical Sciences, Beijing, China; 2 Postdoctoral Research Station, China Academy of Chinese Medical Sciences, Beijing, China

**Keywords:** Condyloma acuminatum, Fu Fang Gang Liu liquid, Network pharmacology, Molecular docking, Transcriptomics

## Abstract

**Background:**

Condyloma acuminatum (CA) is a sexually transmitted disease characterized by the anomalous proliferation of keratinocytes caused by human papillomavirus (HPV) infection. Fu Fang Gang Liu liquid (FFGL) is an effective externally administered prescription used to treat CA; however, its molecular mechanism remains unclear. This study aimed to identify and experimentally validate the major active ingredients and prospective targets of FFGL.

**Methods:**

Network pharmacology, transcriptomics, and enrichment analysis were used to identify the active ingredients and prospective targets of FFGL, which were confirmed through subsequent experimental validation using mass spectrometry, molecular docking, western blotting, and in vitro assays.

**Results:**

The network pharmacology analysis revealed that FFGL contains a total of 78 active compounds, which led to the screening of 610 compound-related targets. Among them, 59 overlapped with CA targets and were considered to be targets with potential therapeutic effects. The protein–protein interaction network analysis revealed that protein kinase B (Akt) serine/threonine kinase 1 was a potential therapeutic target. To further confirm this result, we performed ribonucleic acid sequencing (RNA-seq) assays on HPV 18^+^ cells after FFGL exposure and conducted enrichment analyses on the differentially expressed genes that were screened. The enrichment analysis results indicated that the phosphatidylinositol 3-kinase/protein kinase B (PI3K/Akt) pathway may be a key pathway through which FFGL exerts its effects. Further in vitro experiments revealed that FFGL significantly inhibited the activity of HPV 18^+^ cells and reduced PI3K and Akt protein levels. A rescue experiment indicated that the reduction in cell viability induced by FFGL was partially restored after the administration of activators of the PI3K/Akt pathway. We further screened two active components of FFCL that may be efficacious in the treatment of CA: periplogenin and periplocymarin. The molecular docking experiments showed that these two compounds exhibited good binding activity to Akt1.

**Conclusion:**

FFGL reduced HPV 18^+^ cell viability by inhibiting key proteins in the PI3K/Akt pathway; this pathway may represent an essential mechanism through which FFGL treats CA. Periplogenin and periplocymarin may play a significant role in this process.

**Supplementary Information:**

The online version contains supplementary material available at 10.1186/s12906-023-03960-7.

## Introduction

Condyloma acuminatum (CA) is a sexually transmitted disease with high transmission and recurrence rates that is caused by human papillomavirus (HPV), which belongs to the alpha genus of the papilloma vacuolar virus [1]. Low-risk HPV is the most prevalent type of HPV that causes CA; however, infection with high-risk HPV alone or in combination is also common in CA cases [2]. Patients with high-risk HPV infection also have a higher risk of developing persistent infection and experiencing a longer disease duration than those without high-risk HPV infection [3]. Moreover, high-risk HPV is a key factor associated with certain types of cancer, such as cervical cancer, penile cancer, and head and neck cancers [4].

The most prominent clinical feature of CA is the production of warts caused by the abnormal proliferation of epithelial cells. HPV enters basal cells through tiny wounds and replicates in large quantities in keratinocytes as basal cells differentiate, promoting the abnormal proliferation of epithelial cells and producing warts [5–9]. The classical theory suggests that this process is closely related to the expression of oncoproteins E6 and E7. These oncoproteins interfere with p53 and phosphorylated retinoblastoma tumor suppressor protein (pRb), affecting the regulation of the host cell cycle and apoptosis [10–12]. Furthermore, other signaling pathways are instrumental in the abnormal cellular proliferation caused by HPV infection. A number of studies have reported that the phosphatidylinositol 3-kinase/protein kinase B (PI3K/Akt) pathway is activated in HPV-related diseases and certain cancers [13–15], which can lead to accelerated cell division, metabolic reprogramming, and the inhibition of autophagy [16]. HPV E6 can promote cell division by upregulating the expression of genes in the P13K/Akt pathway and increasing the phosphorylation level of PI3K [17]. HPV E7 markedly increases the activity of Akt in keratinocytes by inactivating the pRb family of proteins [18]. E7 can also activate Akt by promoting Akt phosphorylation at Thr308 and Ser473 residues [19]. One study showed that after intervention with P13K inhibitors, Akt phosphorylation in keratinocytes transfected with HPV 16 was significantly reduced [20].

Traditional Chinese medicine (TCM) has a long history of treating CA. Fu Fang Gang Liu liquid (FFGL) is an effective externally administered prescription for CA that was first described by a well-known Chinese dermatologist, Xu Xian. FFGL comprises the following four herbs: Da Qing Ye *(Isatis indigotica Fort.)*, E Zhu *(Curcuma phaeocaulis Val.)*, Gang Liu *(Periploca sepium Bge.)*, and Bu Gu Zhi *(Psoralea corylifolia L.).* The creation of FFGL was originally based on folk remedies used for the treatment of warts, and it has been used clinically for decades with excellent therapeutic results. Previously, we conducted a clinical study on the topical application of FFGL for CA treatment. The results showed a high wart clearance rate with FFGL treatment, with a lower recurrence rate after treatment than was observed in the podophyllotoxin-treated group [21]. However, the mechanism through which FFGL treats CA requires further elucidation.

TCM compounds have multi-component, multi-target, and multi-pathway characteristics. Network pharmacology is a discipline in which potential targets and mechanisms of drugs are identified by studying the complicated connections between drugs, targets, and disease processes; thus, it can help reveal the mechanisms through which TCM compounds can treat diseases [22–24]. This study’s first aim was to characterize the active ingredients and potential targets of FFGL using network pharmacology to reveal its therapeutic mechanisms. The second aim was to validate the prediction targets using mass spectrometry, molecular docking, RNA sequencing (RNA-Seq), and in vitro experiments.

## Methods

### Network pharmacological analysis

#### Screening of active compounds and determination of action targets 

The compounds comprising FFGL were extracted from the Traditional Chinese Medicine Systems Pharmacology Database and Analysis Platform (TCMSP) [25] and the Encyclopedia of TCM (ETCM) [26]. Pharmacokinetic screening was performed according to the drug absorption, distribution, metabolism, and excretion processes [27, 28]. Considering the therapeutic characteristics of substances for external use, the active ingredients in FFGL with a drug-likeness (DL) ≥ 0.18 were screened out. Most active ingredient targets were identified through the TCMSP, and active ingredients that did not predict target information were supplemented by the PharmMapper [29] and SwissTargetPrediction [30] platforms. A standard normalized fit score > 0.9 was set in PharmMapper, and a probability > 0 was set in SwissTargetPrediction to select potential targets. The predicted target protein information was standardized using the UniProt database [31] to unify genetic names. An Herb-Compound-Target (H-C-T) network was constructed using Cytoscape 3.7.2 [32] to study the relationships between complex components and targets of FFGL.

#### Target collection and potential CA target prediction

Using “condyloma acuminatum” and “genital warts” as search terms, potential targets of CA were mined from the GeneCards database [33] and the DisGeNet database [34], and high-resolution targets larger than the median score were selected. Subsequently, we unified the genetic names using the UniProt database. The intersection of FFGL active component targets and disease targets was then determined. We created an “H-C-T-Disease” (H-C-T-D) network using Cytoscape 3.7.2 to elucidate the connections between FFGL active components and the disease and drug intersection targets.

#### Protein–Protein Interaction (PPI) network construction and analysis

The cross-targets were committed to the Search Tool for the Retrieval of Interacting Genes/Proteins version 11 (STRING11.0) database [35]. The biological species was set to “Homo sapiens,” and the minimum interaction threshold was pegged at > 0.9 (the confidence level). The targets without protein interaction relationships were hidden, and the PPI network diagram was subsequently obtained. Through Cytoscape3.7.2, the PPI network was visualized, and the appropriate degree value was selected to screen the core targets. According to the methods reported in the literature [36], we leveraged the Molecular Complex Detection (MCODE) plugin to extract the clustered networks with highly connected attributes.

### Experimentation section

#### Experimental materials

HeLa cells were obtained from the National Experimental Cell Resource Sharing Platform (Beijing, China). All the TCM tablets used in the FFGL preparation were purchased from Kangmei Pharmaceutical Co., Ltd. (Guangdong, China), including *Isatis indigotica Fort.*, *Curcuma phaeocaulis Val.*, *Periploca sepium Bge.*, and *Psoralea corylifolia L*.

#### Preparation of FFGL

*Isatis indigotica Fort.*, *Curcuma phaeocaulis Val.*, *Periploca sepium Bge.*, and *Psoralea corylifolia L.* herbs were soaked in 1 L of double-steamed water for 30 min according to the clinical dosage and boiled for 1 h. We poured out the liquid medicine and boiled it again with 1 L of double-distilled water for 1 h. The two decoctions were mixed, concentrated into 500 mL of liquid, centrifuged in a molecular sieve at 3,000 kDa to remove macromolecular polysaccharides and polypeptides, and centrifuged at 5,000 × g for 30 min. The centrifuged liquid was concentrated again and lyophilized using a lyophilizer, after which the lyophilized powder was sealed and stored at –80 °C. We dissolved 0.512 g of FFGL extract in 10 mL of sterile double-distilled water into a mother liquor (pH 7.2–7.4), filtered it through a 0.22-micron filter, aliquoted and stored it in a refrigerator at –20 °C, and diluted it to the corresponding concentration in the corresponding medium before use. Next, we characterized the composition of the FFGL lyophilized powder by liquid chromatography–mass spectrometry (LC–MS) at Beijing University of Chinese Medicine. The components identified via LC–MS can be found in Supplementary Table 1.

#### Cell viability assays

We applied the cell counting kit-8 (CCK-8) method to evaluate the effect of FFGL on cell viability according to the manufacturer’s instructions. HeLa (HPV 18^+^) cells were seeded into 96-well plates at a cell density of 3 × 10^4^ cells/mL. To detect the temporal and dose-dependent effects of FFGL on cells, we treated the HeLa cells with different FFGL concentrations (0, 20, 40, 80, 160, 320, 640, 1280, 2560, and 5120 µg/mL) for 24 h, 48 h, and 72 h. Next, we added 10 µL of CCK-8 working solution to the cells, gently shaking the plate for 30 s. The reaction was performed for 3 h at 37 °C in an incubator. Finally, the optical density value was measured using an enzyme marker (TECAN, Männedorf, Switzerland) at an excitation wavelength of 450 nm. Similarly, we treated HeLa cells with the PI3K activator 740 Y-P (GlpBio, Montclair, CA, USA) (0, 12.5, 25, 50, 100, 200 µg/mL) in combination with FFGL (80 µg/mL) for 48 h.

#### RNA-Seq assays

We seeded HeLa cells in 6-well plates at a cell density of 9 × 10^4^ cells/mL the night before treatment. The next day, we treated the cells with 160 μg/mL of FFGL for 48 h. The treated cells were collected, and the total RNA was extracted based on the manufacturer’s instructions. The RNA concentration and purity were evaluated using an Agilent 2100 Bioanalyzer (Agilent, Palo Alto, CA, USA). Quality-checked mRNAs (RIN ≥ 7 and 28S/18S ≥ 0.7) were added to Oligo (dT) magnetic beads for mRNA isolation, mRNA fragmentation, and cDNA synthesis. The synthesized cDNA fragments were subjected to end-repair, and a single ‘A’ nucleotide was added to the 3’ ends of the blunt fragments. After adaptor ligation, the products were amplified by PCR to generate libraries. The PCR products were denatured to single-stranded loops and then sequenced on a BGISEQ-500 platform (Beijing Genomics Institute, Beijing, China). The raw sequencing data were filtered using SOAPnuke (v1.5.6) software to obtain clean data. Subsequently, the clean data were aligned to the University of California, Santa Cruz (UCSC) human reference genome (hg38) using Bowtie2 (v2.3.4.3) software. Gene expression quantification was performed using RSEM (v1.3.1) software. The statistics describing the RNA quality after filtering can be found in Supplementary Table 2. Detailed RNA-seq data can be found in the National Center for Biotechnology Information (NCBI) Gene Expression Omnibus (GEO) (GSE223380). A |Log2Fold Change|> 1.5 and a *q*-value < 0.05 were used as the screening criteria to identify differentially expressed genes (DEGs). Volcano maps of the DEGs were generated using HiPlot as previously described [37]. R software (v 4.1.2) and the Kyoto Encyclopedia of Genes and Genomes (KEGG) Orthology-Based Annotation System (KOBAS) 3.0 [38] were used to conduct the bioinformatics analysis of the DEGs.

#### Bioinformatics analysis

The clusterProfiler packge (v 4.0.3) [39] in R software was used to conduct the Gene Ontology (GO) enrichment analysis, and the enrichment terms were visualized using tree network diagrams. KEGG [40–42] enrichment analysis of DEGs was performed using KOBAS 3.0 and visualized using HiPlot. The Benjamini–Hochberg procedure was used to correct the p-values. Corrected *p*-values < 0.05 was considered a significant enrichment.

#### Western blot analysis

We seeded HeLa cells in 6-well plates at a cell density of 3 × 10^5^ cells/mL, then we treated the cells with 160 μg/mL of FFGL at different time points. Subsequently, the HeLa cells were lysed in radioimmunoprecipitation assay buffer for 30 min on ice. After extracting the total protein content, we quantified it using a bicinchoninic acid (BCA) Protein Concentration Assay Kit (Solarbio, Beijing, China) and separated the proteins via 10% or 12% sodium dodecyl sulfate–polyacrylamide gel electrophoresis. The separated proteins were then transferred to a polyvinylidene fluoride membrane. Next, they were blocked with 5% skim milk for 45 min and incubated overnight with the following primary antibodies: anti-PI3K (1:1000, CST, Danvers, MA, USA) and anti-Akt (1:1000, CST, USA). Subsequently, the membrane was washed thrice with tris-buffered saline (TBS) containing 0.1% Tween-20 and incubated with a secondary antibody (1:5000, CST, USA) for 1 h. The membrane was exposed to an enhanced chemiluminescence reagent (Millipore, Billerica, MA, USA) and then imaged using an infrared laser scanning instrument (Analytik Jena, Jena, Germany). Protein bands were scanned and analyzed using ImageJ software for optical densitometry for the semi-quantitative determination of the protein expression levels. The experiment was repeated three times.

### Molecular docking

Molecular docking techniques were used to analyze the interactions between the key components in FFGL and the key targets of CA. The two-dimensional structures of the key components were obtained from structure data files (SDFs) by importing them into the PubChem database [43]; the SDF structures of these compounds were imported into the Schrödinger molecular docking software and preprocessed using the LigPre module for later molecular docking preparation. The three-dimensional structures of the key targets were downloaded from the Research Collaboratory for Structural Bioinformatics Protein Data Bank (RCSB PDB) database [44], and those with ligands of human origin were selected, downloaded, and imported into the Schrödinger molecular docking software. The proteins were preprocessed using the Protein Preparation module, which included the deletion of water molecules, hydrogenation, residue complementation, and charge assignment. The active pockets for molecular docking were defined by the ligands that came with the crystals, and the docking boxes were generated by directly selecting the ligands in the crystals. Screening for standard precision patterns was conducted for the docking of key components of the compounded solution into the defined active pockets, and the docking results were visualized using PyMol V 2.1.0.

### Statistical Analysis

Values are expressed as the mean ± standard error of the mean, unless otherwise noted. The statistical significance of differences between the control and treatment groups was assessed using unpaired, two-tailed Student’s *t*-tests (GraphPad Prism). All comparisons were made relative to untreated controls, and *P* < 0.05 was considered statistically significant. Unless otherwise stated, three independent experiments were conducted for each variable.

## Results

### Construction of the H-C-T network

**Fig. 1:**
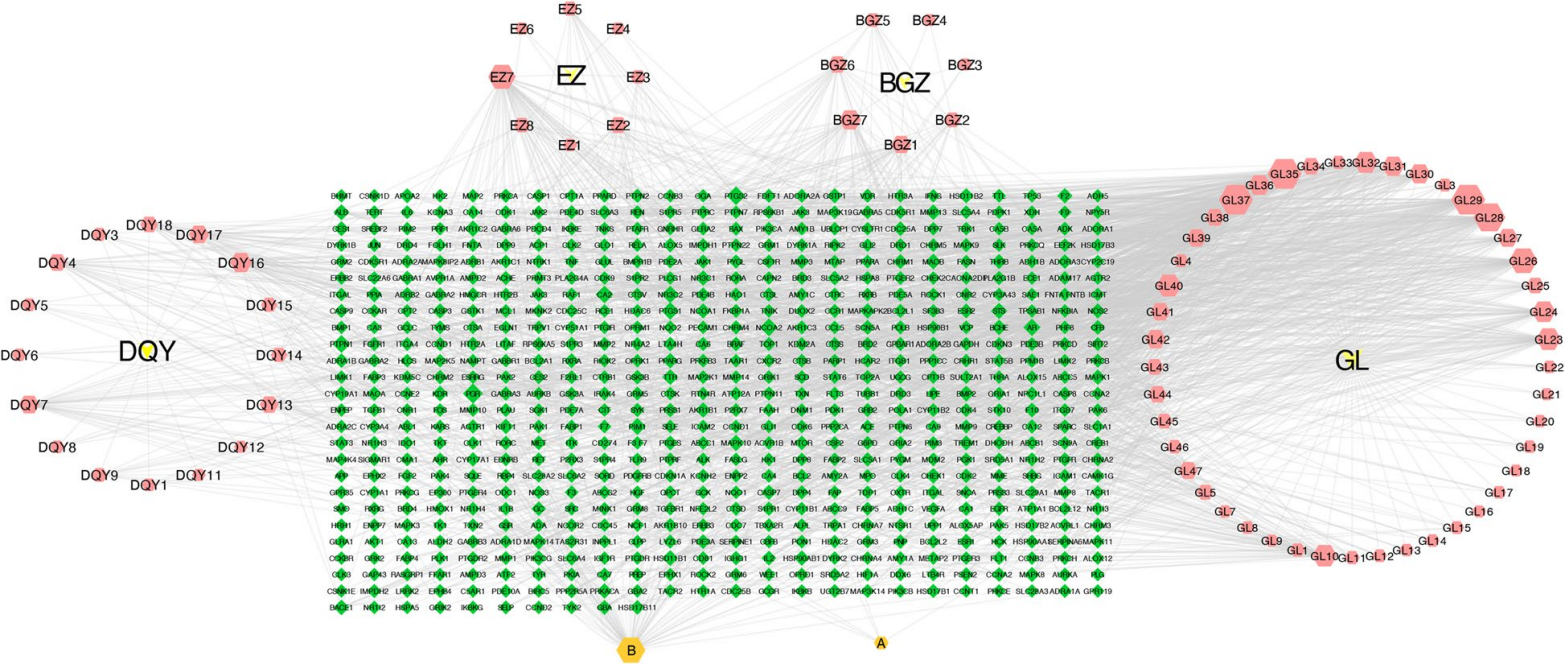
Herb-compound-target network of Fu Fang Gang Liu liquid (FFGL). The yellow “Vs” represent the herbs present in FFGL. Pink hexagons represent active compounds in each herb, and the orange hexagons labeled **A** and **B** represent active compounds shared by the two herbs *Periploca sepium Bge.* and *Isatis indigotica Fort.* Green diamonds correspond to related targets. Active compounds are shown as entry names, such as “GL15” from Gang Liu. In this network, the degree of each node represents the number of lines connected with the nodes; core nodes are screened according to the characteristics of network topology, such as the node degree value

Seventy-eight active ingredients were identified in FFGL, including 18 from *Isatis indigotica Fort.*, seven from *Psoralea corylifolia L.*, 47 from *Periploca sepium Bge.*, and eight from *Curcuma phaeocaulis Val.* Two of the components, poriferast-5-en-3beta-ol and beta-sitosterol, were common to *Isatis indigotica Fort.* and *Periploca sepium Bge.* A total of 610 targets associated with 78 active compounds in FFGL were obtained after removing duplicates, including 163 for *Isatis indigotica Fort.*, 114 for *Curcuma phaeocaulis Val.*, 105 for *Psoralea corylifolia L.*, and 539 for *Periploca sepium Bge.* The “H-C-T” network of FFGL was constructed and analyzed using Cytoscape to investigate further interactions between components and targets in FFGL (Fig. 1). The “H-C-T” network consisted of 692 nodes and 1,915 edges. Perlatolic acid, periplocymarin, and neridienone A had the greatest number of targets in the H-C-T network, with 100 targets for each component. Among the remaining compounds, periplogenin, beta-sitosterol, hederagenol, and bisdemethoxycurcumin had the next highest numbers of targets, with 90, 75, 72, and 48, respectively. Further details can be found in Supplementary Table 3.

### Acquisition of potential targets for CA treatment

The GeneCards and the DisGeNet databases were used to obtain potential targets for CA. After removing duplicate targets contained in both databases, we obtained 346 CA-related targets (Supplementary Table 4). To explore the key CA targets of the active components of FFGL, we overlapped the targets regulated by the active components in FFGL with the CA-related targets, yielding 59 core targets (Supplementary Table 5).

### Construction of the H-C-T-D network

**Fig. 2:**
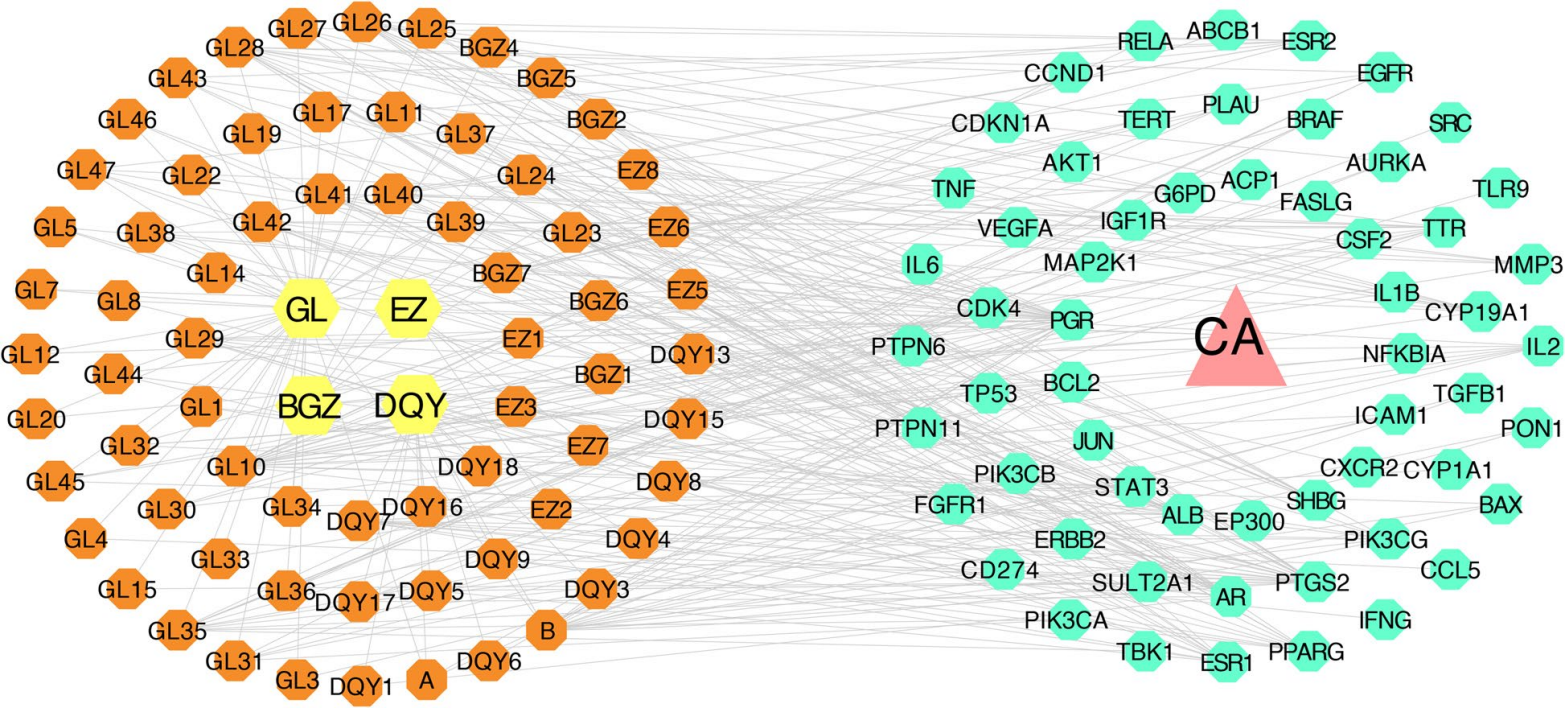
Herb-compound-target-disease network. The pink triangle represents the disease condyloma acuminatum (CA). The yellow hexagons represent the herbs in Fu Fang Gang Liu liquid (FFGL). The orange octagons represent the main active compounds contained in the core herbs of FFGL. The green octagons typify the potential targets. Core components are screened according to the node degree value

The H-C-T-D network was constructed using Cytoscape. The network comprised 132 nodes and 340 edges (Fig. 2). The following top 10 compounds were filtered according to the degree value: ursolic acid, beta-sitosterol, periplogenin, hederagenol, periplocymarin, amyrin, indirubin, corylifolinin, 3’-methoxysecoisolariciresinol, and perlatolic acid (Table 1). Detailed information is provided in Supplementary Table 6.

**Table 1:** The top 10 compounds according to the degree value

Compound	Herb	Molecular formula	Structure	Molecular weight (g/mol)	DL	Degree
ursolic acid	GL	C_30_H_48_O_3_		456.7	0.75	22
beta-sitosterol	DQY/GL	C_29_H_50_O		414.7	0.75	17
periplogenin	GL	C_23_H_34_O_5_		390.5	0.74	15
hederagenol	GL	C_30_H_48_O_4_		472.7	0.74	14
periplocymarin	GL	C_30_H_46_O_8_		534.7	0.67	12
amyrin	GL	C_30_H_50_O		426.7	0.76	11
indirubin	DQY	C_16_H_10_N_2_O_2_		262.26		9
corylifolinin	BGZ	C_20_H_20_O_4_		324.4		9
3’- methoxyseco isolariciresinol	GL	C_21_H_28_O_7_		392.5	0.38	9
perlatolic acid	GL	C_25_H_32_O_7_		444.5	0.54	9

*GL *Gang Liu, *BGZ *Bu Gu Zhi, *DQY *Da Qing Ye

### PPI network construction and clustering analysis of core targets

**Fig. 3:**
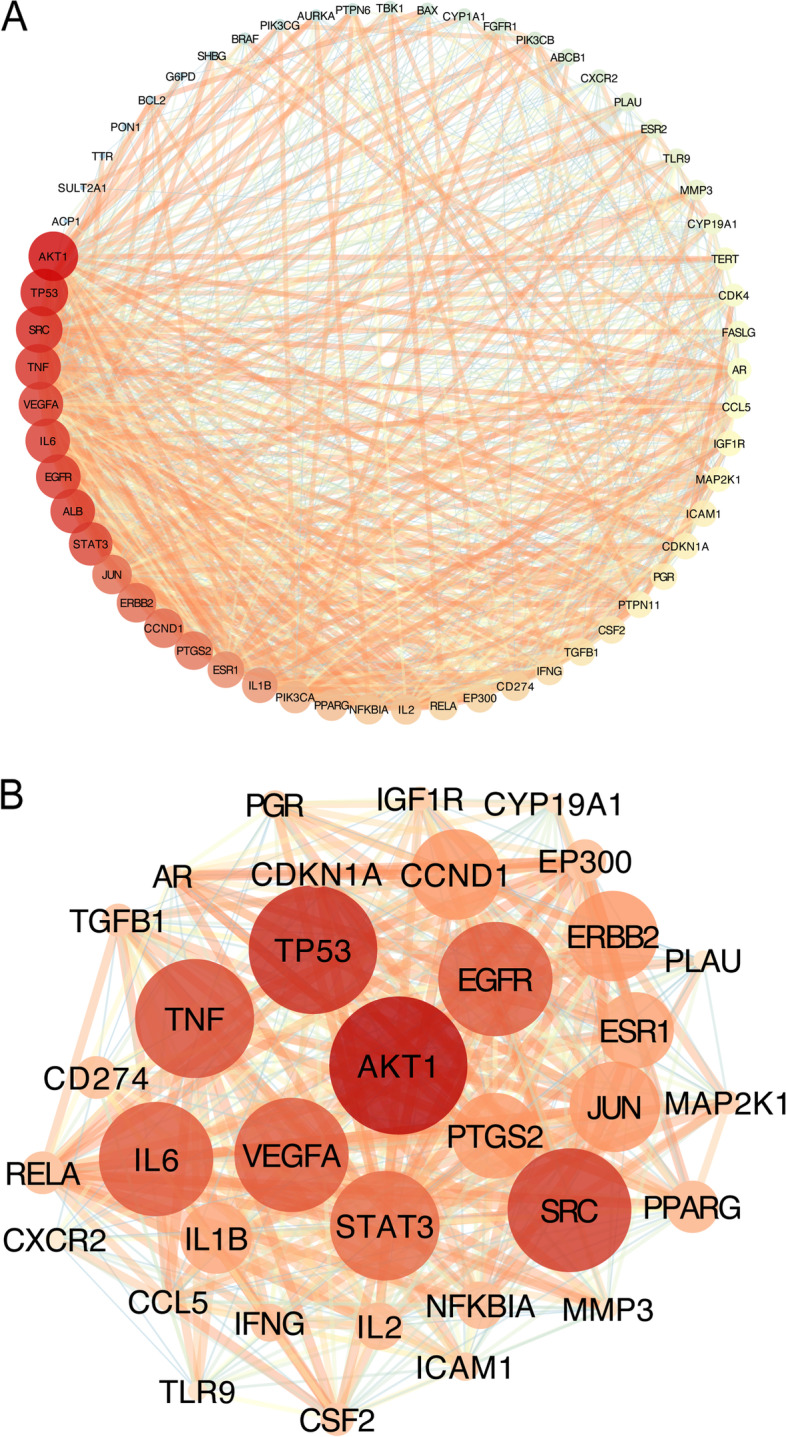
Protein–protein interaction (PPI) network of core targets for Fu Fang Gang Liu liquid (FFGL) related to condyloma acuminatum (CA). Each node represents a protein target, and each line represents the interaction between two nodes. The darker and larger nodes are related to a higher degree value of the target. Thicker lines indicate a larger correlation. Panel (**A**) is the PPI network of FFGL in the treatment of CA. Panel (**B**) is the cluster detected in the FFGL-CA PPI network by Molecular Complex Detection

The 59 potential core targets of FFGL for CA that we acquired were imported into the STRING database to construct a PPI network. Next, we imported the constructed PPI network into Cytoscape for further visualization and in-depth analysis. This network included 59 nodes and 804 edges (Fig. 3A). Each node represents a protein, and the degree value indicates the number of lines connected to one node and is used as a measure of the importance of each node. The size of the nodes and color hue are proportional to the degree value. The edges of the diagram represent the relationship between each node, and the number of lines is proportional to the degree of association between two nodes.

In the PPI network, the average degree value of each node was 27.25, with 31 nodes having a greater-than-average degree value, including AKT1, TP53, SRC, TNF, EGFR, ALB, IL6, VEGFA, STAT3, CCND1, ERBB2, JUN, PTGS2, ESR1, IL1B, PIK3CA PPARG, NFKBIA, IL2, EP300, RELA, CD274, IFNG, TGFB1, CSF2, PTPN11, ICAM1, PGR, CDKN1A, IGF1R, and MAP2K1. These 31 targets revealed the preliminary relationship between potential protein targets of FFGL for the treatment of CA.

The MCODE plugin in Cytoscape was used to discover the crucial clustering of functional modules in the PPI network, and a K-core of 4 was set as the threshold to obtain the clustering of one functional module (Fig. 3B). This clustering network consisted of 35 nodes and 463 edges, with a score of 27.235. The top 10 nodes according to the degree size were AKT1, TP53, SRC, TNF, EGFR, VEGFA, IL6, STAT3, CCND1, and JUN. The core node was Akt1, a member of the serine/threonine protein kinase subfamily that is highly expressed in many tumors and HPV-related diseases [45, 46]. In this clustering module, other nodal proteins are directly or indirectly involved in Akt1 activation, such as the extracellular-associated factors, VEGFA and EGFR, which bind to dimerize the protein [47]. Akt1 modulates events related to cell death and senescence by enhancing the ubiquitinated degradation of p53 promoted by MDM2 [48]. These findings indicate that Akt1, the core node, is involved in the regulation of biological processes such as cell proliferation, differentiation, and migration by connecting with other cytokines in the whole cluster. This analysis of the clustering network suggests that AKT1 is an essential target of FFCL for the treatment of CA. Detailed information is provided in Supplementary Tables 7 and 8.

### FFGL can significantly reduce the viability of HPV 18^+^ cells

**Fig. 4:**
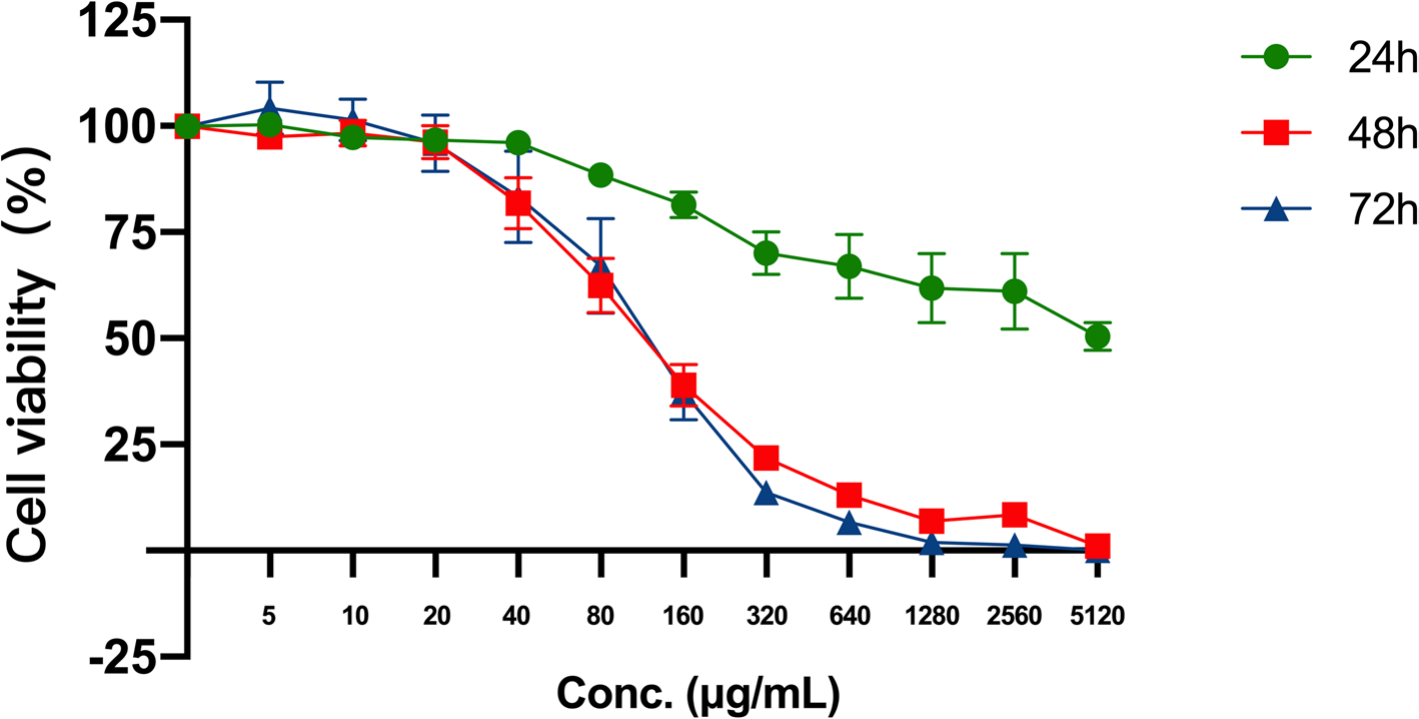
Fu Fang Gang Liu liquid (FFGL) significantly inhibits the cell viability of human papillomavirus 18 + cells. HeLa cells were treated with FFGL (0, 20, 40, 80, 160, 320, 640, 1280, 2560, or 5120 μg/mL) for 24, 48, and 72 h. Cell viability was measured using a cell-counting kit-8 assay (*n* = 3)

To further clarify the effect of FFGL on HPV 18^+^ cells, HeLa cells containing the entire HPV 18 genome were used. The effect of FFGL on the viability of HeLa cells was examined after 24-, 48- and 72-h interventions. We found that FFGL significantly inhibited the viability of HeLa cells, and the effect was both time- and dose-dependent (Fig. 4).

### RNA-Seq analysis

**Fig. 5:**
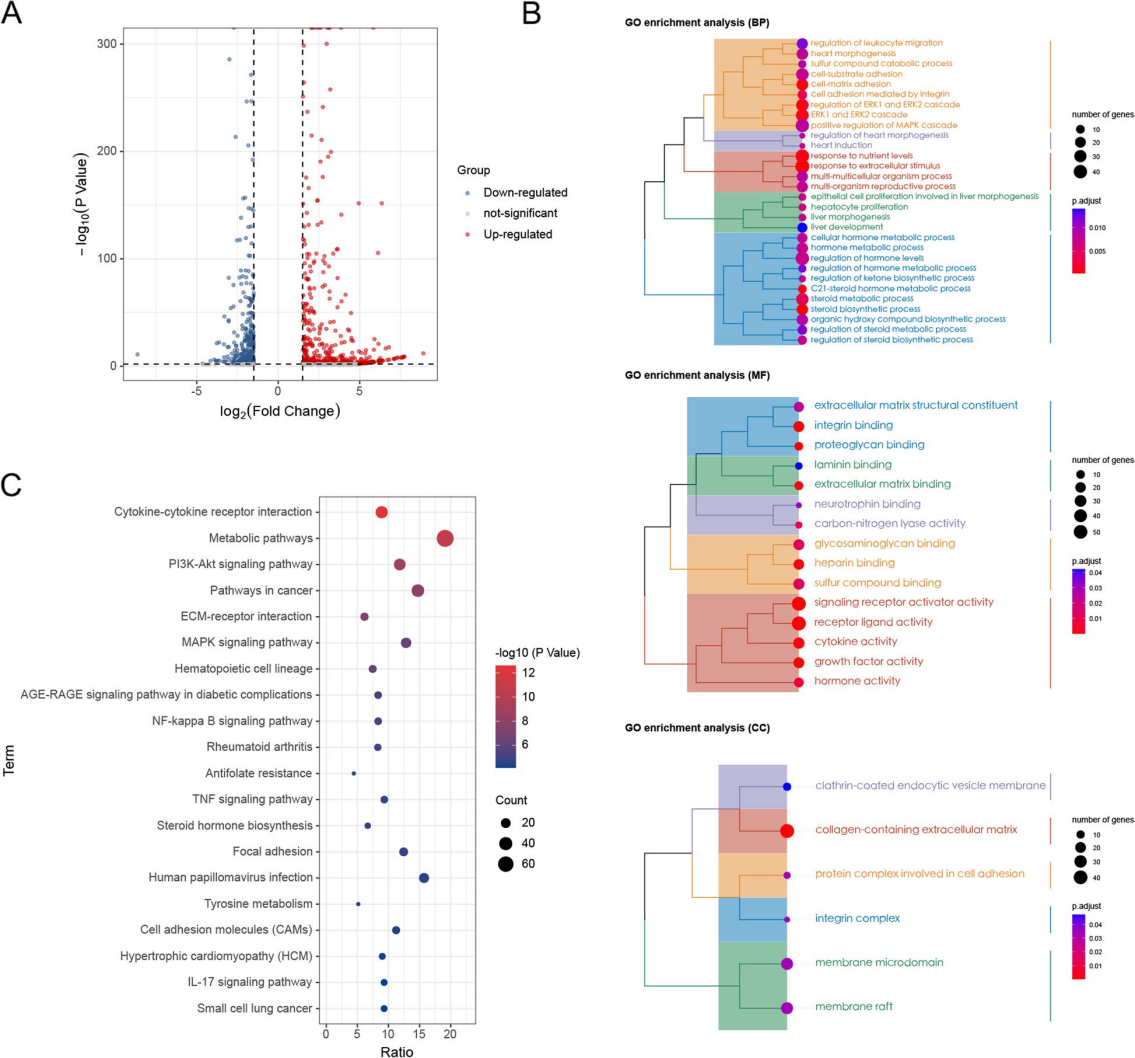
Identification of differentially expressed genes (DEGs) and enrichment analysis. **A** Gene expression data presented as a volcano plot. **B** Tree network diagram of the Gene Ontology enrichment results. Biological process (BP), molecular function (MF), and cellular component (CC) are shown from top to bottom. **C** Results of the Kyoto Encyclopedia of Genes and Genomes (KEGG) enrichment analysis of DEGs

To further explore the mechanism of action, we collected and sequenced RNA from HeLa cells from the control group and those treated with 160 μg/mL FFGL for 48 h. Based on the screening criteria, we screened 883 DEGs, 354 of which were upregulated and 529 of which were downregulated. Volcano plots of all gene expression data were created (Fig. 5A).

### GO and KEGG enrichment analyses

To explore the mechanism through which FFGL inhibits the proliferation of HPV^+^ cells in more depth, we performed GO enrichment analysis of DEGs using the clusterProfiler package and KEGG enrichment analysis of DEGs using KOBAS 3.0, then visualized the results using HiPlot. The GO enrichment analysis consisted of the following three components: biological process (BP), molecular function (MF), and cellular component (CC). The BP enrichment analysis showed that DEGs were mainly enriched in processes related to the response to extracellular stimuli, steroid biosynthesis, and ERK1 and ERK2 signaling cascades. The MF enrichment analysis showed that DEGs were mainly enriched in processes related to receptor–ligand activity, signaling receptor activator activity, and integrin binding. The CC enrichment analysis showed that the DEGs were mainly involved in collagen-containing extracellular matrices, integrin complexes, and protein complexes involved in cell adhesion (Fig. 5B). Detailed information on enriched pathways is shown in Supplementary Table 9.

The KEGG enrichment analysis revealed that the DEGs were mainly enriched in processes related to cytokine-cytokine receptor interactions, metabolic pathways, PI3K-Akt signaling pathways, and cancer pathways. Figure 5C presents the top 20 of these enriched pathways.

### FFGL suppresses the PI3K/Akt pathway

**Fig. 6:**
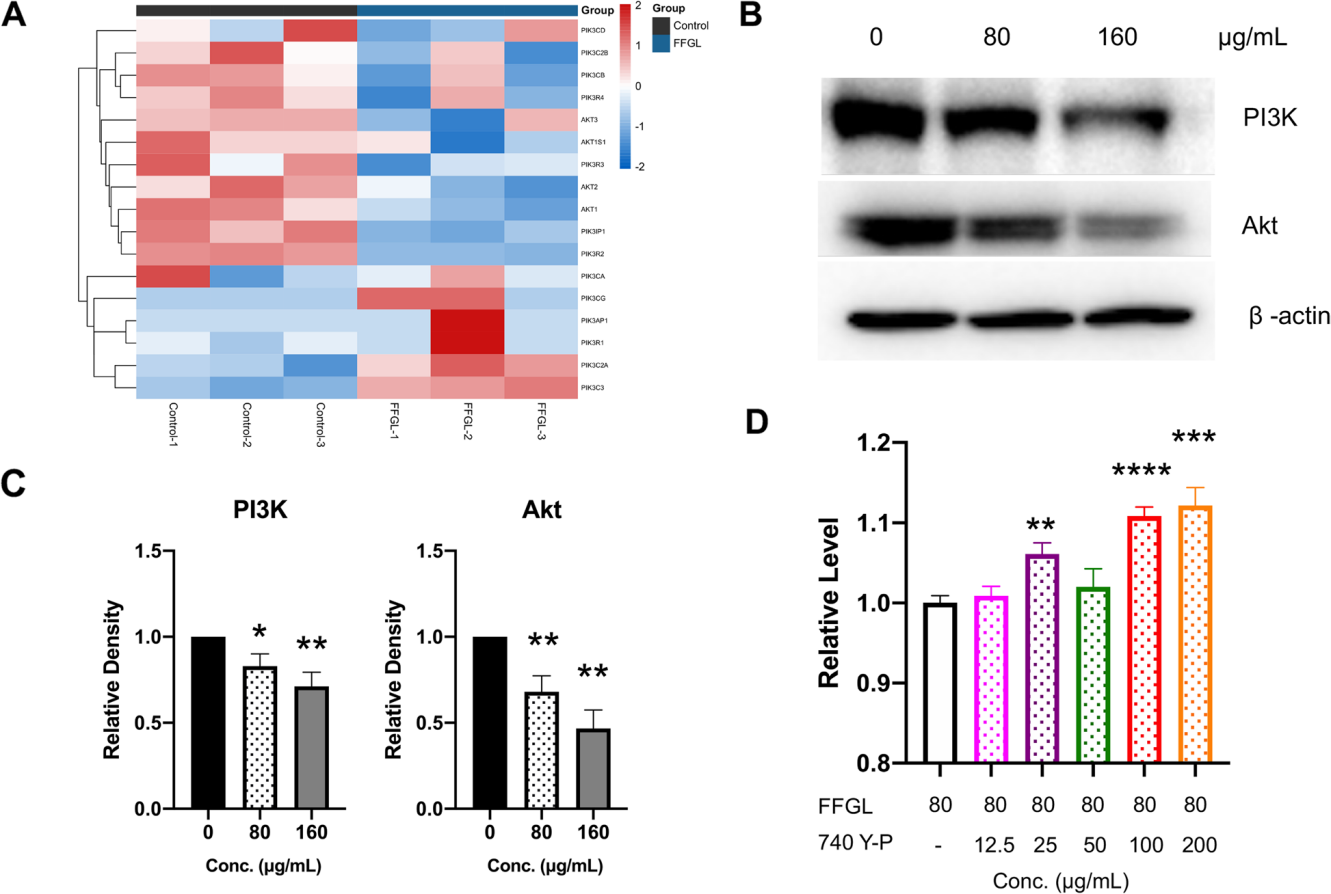
Fu Fang Gang Liu liquid (FFGL) inhibits the phosphatidylinositol 3-kinase/protein kinase B (PI3K/Akt) pathway. **A** We extracted the transcripts per kilobase million values of PI3K- and Akt-related genes from the RNA sequencing results and observed that the expression of most genes was reduced. **B** **C** Cells were treated with FFGL (160 μg/mL) for 12 h, and the expression of PI3K and Akt proteins was analyzed by western blotting. β-Actin was used as an internal control. **D** Cells were treated with FFGL (80 μg/mL) with various concentrations of 740 Y-P for 48 h. Cell viability was measured using the cell-counting kit 8 assay. Data are presented as the mean ± standard error of the mean (*n * =  3). ^*^ P < 0.05, ^**^ P < 0.01, ^***^ P < 0.005, ^****^ P < 0.001 represent significant differences compared with the values in the untreated control group

Based on the network pharmacology and transcriptome data, we suggest that FFGL may inhibit the proliferation of HPV 18^+^ cells by suppressing the PI3K/Akt signaling pathway. We calculated the expression of PI3K- and Akt-related genes in the transcriptome data and found that the fragments per kilobase of transcript per million mapped reads (FPKM) values of most PI3K- and Akt-related genes were reduced (Fig. 6A). We validated these results by western blot analysis, which revealed that the protein levels of PI3K and Akt in HPV 18^+^ cells were significantly reduced after 12 h of FFGL exposure (Fig. 6B, 6C). Furthermore, we administered 740 Y-P (an agonist of the PI3K/Akt pathway) in combination with FFGL in HPV 18^+^ cells and found that the agonist partially attenuated the inhibition of cell viability induced by FFGL (Fig. 6D).

### Molecular docking verification of FFGL active compounds and core protein targets

**Fig. 7:**
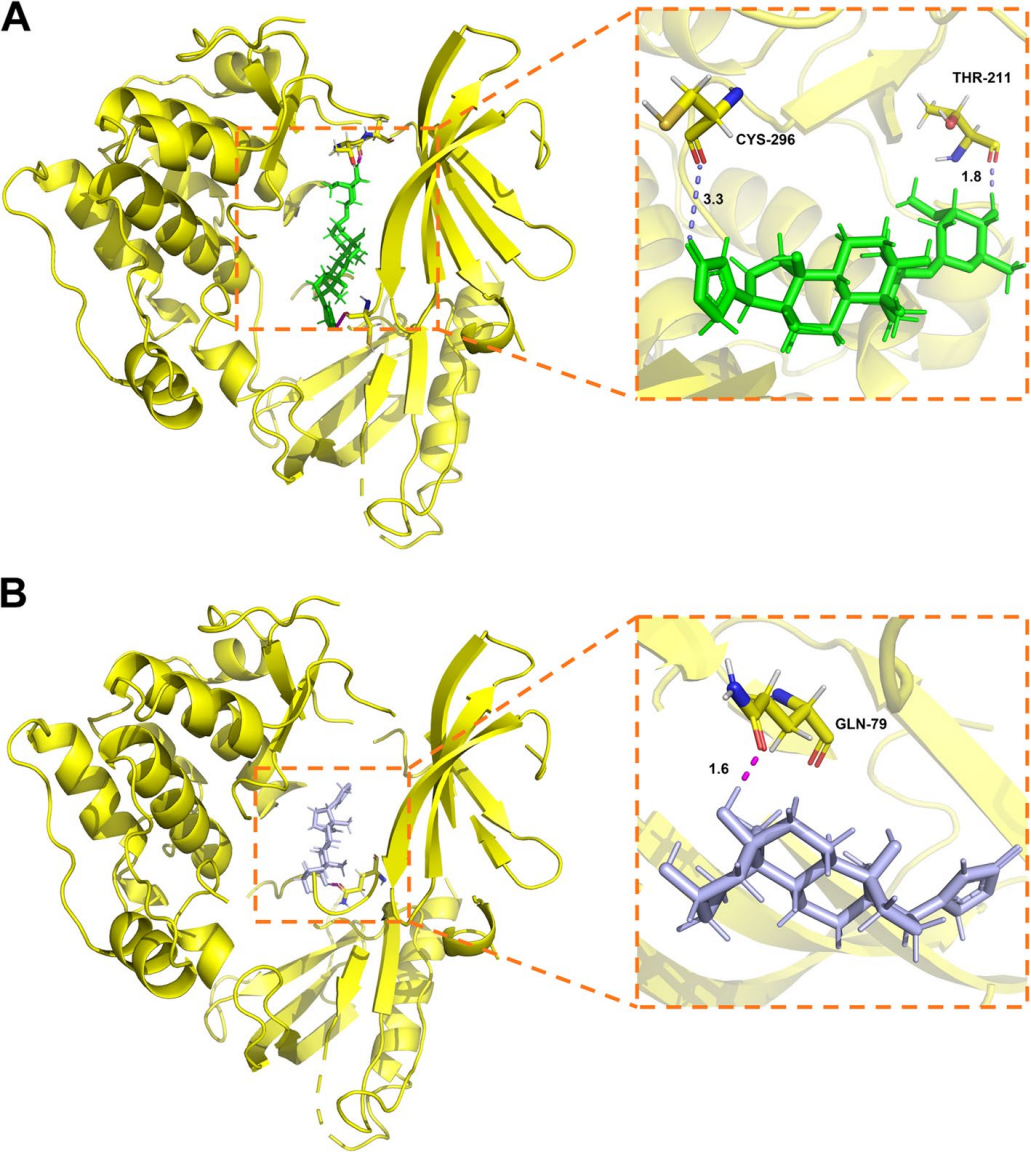
Molecular docking diagram of periplocymarin (**A**) and periplogenin (**B**) with AKT serine/threonine kinase 1

We overlapped the compounds identified by high-resolution mass spectrometry with the top 10 compounds predicted by network pharmacology, which led to the screening of two shared compounds, periplogenin and periplocymarin. Next, Schrödinger molecular docking software was used to dock the two compounds into the core protein Akt1 to conduct the binding free energy analysis. We found that both molecules could access the activity pocket of Akt1. Periplocymarin formed hydrogen bonds with residues CYS-296 and THR-211 of Akt1 at distances of 3.3 A and 1.8 A, respectively (Fig. 7A). Moreover, periplogenin formed a hydrogen bond with residue GLN-79 of Akt1 at a distance of 1.6 A (Fig. 7B). The results of the molecular docking analysis showed that periplocymarin and periplogenin had negative binding free energy to Akt1, indicating that both molecules can spontaneously bind to the Akt1 protein. Further analysis of the results showed that their binding energies were both less than –5 kJ/mol, demonstrating their strong binding affinity (Table 2).

**Table 2:** Molecular docking scores

Target	Molecule name	Molecular formula	Structure	Molecular weight (g/mol)	Docking score (kcal/mol)
Akt1	Periplocymarin	C_30_H_46_O_8_		534.7	-6.169
	Periplogenin	C_23_H_34_O_5_		390.5	-6.142

## Discussion

**Fig. 8:**
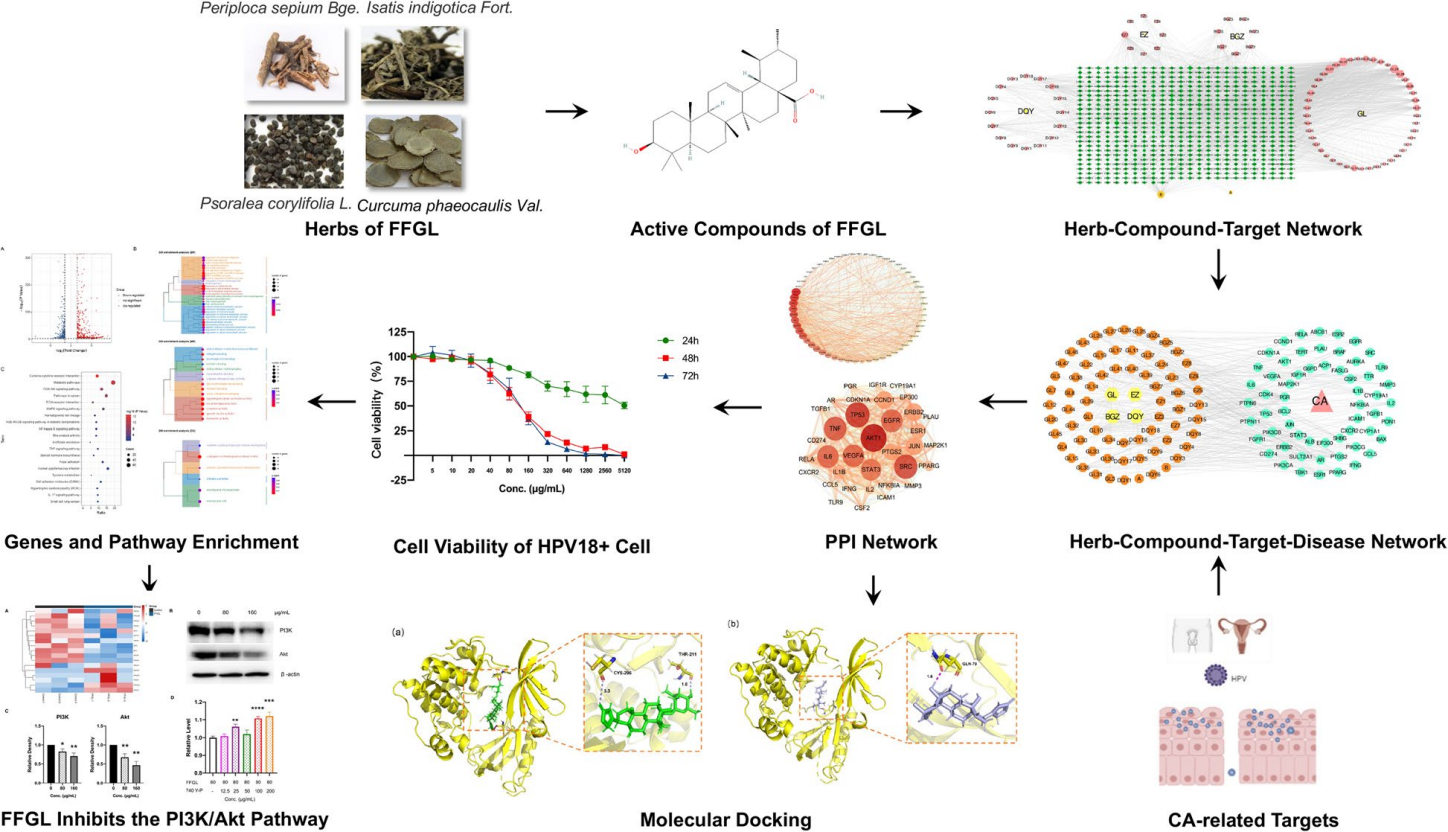
Flow chart to investigate the mechanisms of action of Fu Fang Gang Liu (FFGL) in the treatment of condyloma acuminatum

CA is the most common sexually transmitted disease caused by HPV infection, affecting 160–289 people per 100,000 worldwide [49, 50]. Its high rates of transmission and recurrence result in a huge psychosocial and public health burden [1, 51]. Hyperproliferation of keratinocytes is the main feature of CA, a process that is closely associated with HPV genome-regulating events, such as those related to epithelial cell proliferation and differentiation, immunity, and energy metabolism [7, 52, 53]. Current treatments for CA include immunomodulators, such as imiquimod and interferon; cytotoxic drugs, such as 5-fluorouracil; physiotherapy; photodynamic therapy; cryotherapy; and surgery [50]. However, all of these therapies have limitations. Chinese herbal medicines for the treatment of CA and HPV infection have received increasing attention due to their superior clinical efficacy, fewer side effects, and better safety profile, complementing the limitations of current treatment modalities [54]. In previous clinical studies, we found that FFGL had significant efficacy against CA [21]. To further explore the potential mechanism of FFGL, we used network pharmacology, molecular docking, transcriptomics, and in vitro experiments. Figure 8 shows a flow chart of the experiments conducted in this study.

We selected 59 predicted FFGL targets for CA. Further analysis revealed that these potential core targets were concentrated in Akt1 signaling-related pathways. Akt1 belongs to the Akt protein kinase family and plays a role in HPV-mediated hyperproliferation of keratinocytes [45, 55, 56]. It is overexpressed in many cancers and diseases caused by HPV, including CA. A bioinformatics analysis of CA and normal tissues indicated that the PI3K/Akt pathway was significantly enriched in CA tissues [57]. Further studies revealed that the expression levels of PI3K and p-Akt were dramatically higher in CA tissues compared to those in normal tissues [58, 59]. Moreover, clinically effective 5-aminolevulinic acid photodynamic therapy (ALA-PDT) for CA can inhibit the PI3K/Akt pathway, and it has been proven that inhibition of this pathway can decrease the proliferation rate and increase autophagy and apoptosis of HPV 18^+^ HeLa cells [60]. These studies suggest that Akt1 may be a key target protein for CA treatment.

To further validate this result, we performed a transcriptome analysis of HPV 18^+^ cells after FFGL intervention. We found that the DEGs were mainly enriched in KEGG pathways such as those related to cytokine-cytokine receptor interaction, PI3K-Akt signaling, and pathways in cancer. In conjunction with our previous network pharmacology findings, we suggest that FFGL may act on HPV 18^+^ HeLa cells by interfering with the intracellular PI3K/Akt signaling pathway. Next, we determined the expression of PI3k and Akt-related genes in the transcriptome data and found that the FPKM values of most PI3K- and Akt-related genes were reduced. We also performed western blot experiments, which showed that FFGL significantly reduced PI3K and Akt protein levels in HeLa cells and inhibited the PI3K/Akt pathway. Finally, we found that the administration of activators of the PI3K/Akt pathway could partially attenuate the reduction in cell viability induced by FFGL. In summary, we concluded that FFGL can inhibit the viability of HPV 18^+^ cells through the PI3K/Akt pathway.

The network pharmacology analysis identified 78 FFGL active ingredients from the TCMSP and ETCM databases. Based on the H-C-T network topology analysis and the results of the HPLC–MS experiments, we found that the core compounds contained periplogenin and periplocymarin, which may play an essential role in resistance to HPV infection and in the inhibition of the abnormal activation of the PI3K/Akt pathway in cells. Periplogenin and periplocymarin are cardiac glycosides contained in *Periploca sepium Bunge*, and they possess potent antitumor activity in addition to their ability to prevent heart failure and their antirheumatic effects [61–64]. Periplogenin was first isolated from a chloroform extract of *Periploca sepium Bunge* by Itokawa [65], and the compound markedly inhibited the growth of the malignant ascites cells of the S180 cell line. Recent studies have shown that periplogenin can target signal transducer and activator of transcription 3 (STAT3) to inhibit the growth of squamous cell carcinoma both in vivo and in vitro [66]. A network pharmacology study [67] suggested that periplogenin may inhibit nasopharyngeal carcinoma through the PI3K-Akt pathway. Similarly, periplocymarin also exerts antitumor activity [68] and can inhibit the proliferation of PC3 and U937 cell lines; it can also induce cell arrest in the G2/M phase in U937 cells without causing extensive cell death, making U937 cells sensitive to non-apoptotic doses of apoptosis-inducing ligands [69]. A pharmacokinetic study of periplocymarin showed that it is highly permeable, without p-glycoprotein efflux and cytochrome P450 inhibition, suggesting high bioavailability and low cytotoxicity, with great potential for drug development [70]. Recent studies have found that periplocymarin can induce apoptosis in colorectal cancer cells by inhibiting the PI3K-Akt pathway [71]. Accordingly, our molecular docking results showed that the molecular docking binding energy of both drugs to Akt1 was negative and less than –5, indicating that they could easily bind to the active pocket site of Akt1. These results suggest that FFGL may have reduced HeLa cell viability by inhibiting the PI3K-Akt pathway, an effect mediated by the key components periplogenin and periplocymarin.

## Conclusion

In conclusion, FFGL can inhibit the viability of HPV 18^+^ cells by inhibiting key proteins involved in the PI3K/Akt pathway; this may partially represent the mechanism through which FFGL is efficacious in treating CA. Periplogenin and periplocymarin may play a significant role in this process.

## Supplementary Information

**Additional file 1.**

**Additional file 2.**

**Additional file 3.**

**Additional file 4.**

**Additional file 5.**

**Additional file 6.**

**Additional file 7.**

**Additional file 8.**

**Additional file 9.**

**Additional file 10.**

## Data Availability

The datasets used and/or analysed during the current study are available from the corresponding author on reasonable request.

## Abbreviations

CA: Condyloma acuminatum
HPV: Human papillomavirus
pRb: Phosphorylated retinoblastoma tumor suppressor protein
FFGL: Fu Fang Gang Liu
AKT1: AKT serine/threonine kinase 1
PI3K/Akt: Phosphatidylinositol 3-kinase/protein kinase B
TCM: Traditional Chinese medicine
RNA-Seq: RNA sequencing
TCMSP: Traditional Chinese Medicine Systems Pharmacology Database and Analysis Platform
ETCM: Encyclopedia of TCM
DL: Drug-likeness
H-C-T: Herb-Compound-Target
H-C-T-D: Herb-Compound-Target-Disease
PPI: Protein–Protein Interaction
STRING: Search Tool for the Retrieval of Interacting Genes/Proteins
LC–MS: Liquid chromatography–mass spectrometry
MCODE: Molecular Complex Detection
CCK-8: Cell Counting Kit-8
NCBI: National Center for Biotechnology Information
GEO: Gene Expression Omnibus
GO: Gene Ontology
DEGs: Differentially expressed genes
BCA: Bicinchoninic acid
TBS: Tris-buffered saline
SDF: Structure data file
RCSB PDB: Research Collaboratory for Structural Bioinformatics Protein Data Bank
KEGG: Kyoto Encyclopedia of Genes and Genomes
BP: Biological process
MF: Molecular function
CC: Cellular component
ALA-PDT: 5-Aminolevulinic acid photodynamic therapy
STAT3: Signal transducer and activator of transcription 3
