# Supplementary material for: Mechanisms of action of Fu Fang Gang Liu liquid in treating condyloma acuminatum by network pharmacology and experimental validation
Source: BMC Complement Med Ther. 2023 Apr 20;23:128. doi: 10.1186/s12906-023-03960-7 (PMC10116837; doi:10.1186/s12906-023-03960-7)

Supplementary Figure

The Uncropped immune blot data of PI3K and Akt proteins in FFGL-exposed Hela. The red arrow indicates the location of the target bands. Edges are not visible in some blots because other parts of those blots were used for another experiment.

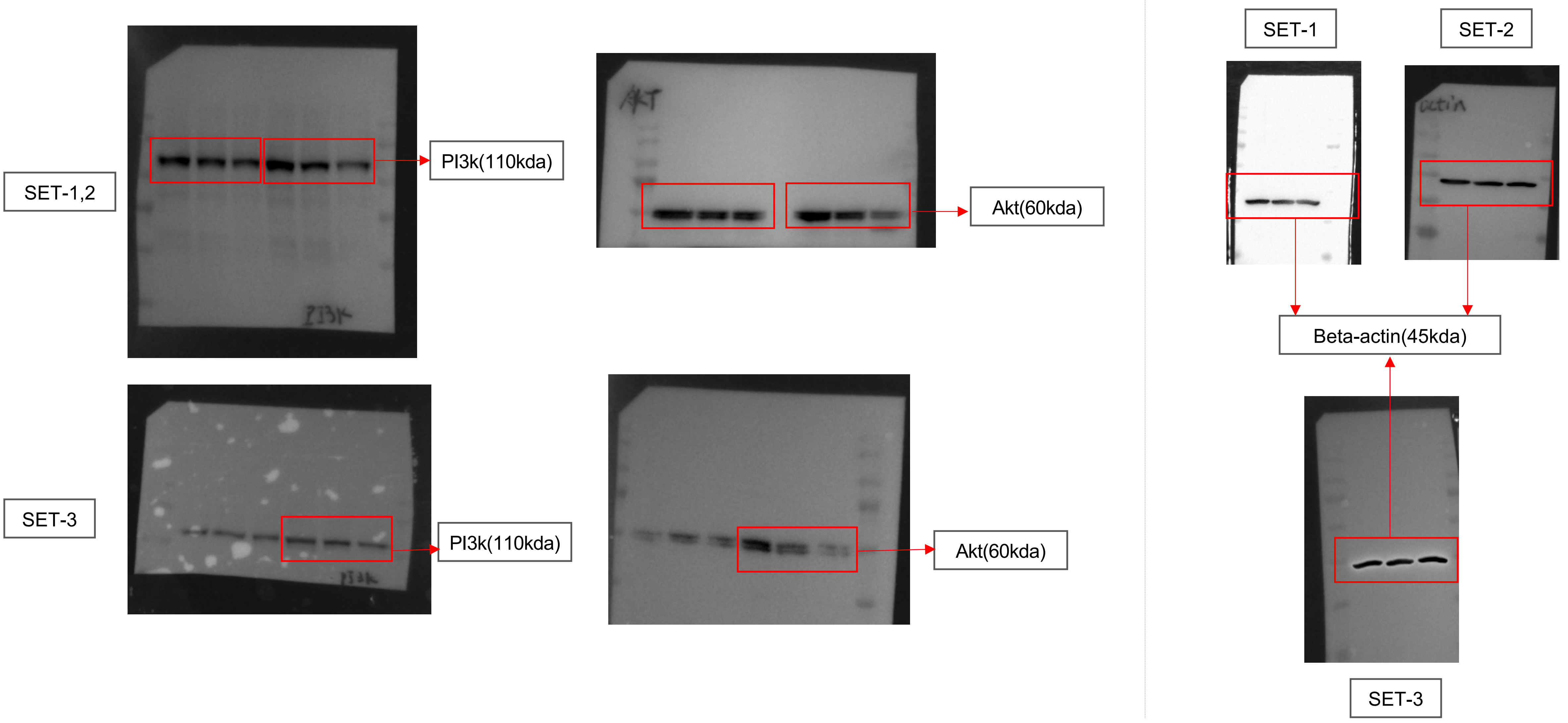

Supplement: Supplementary file 10 — Additional file 10. [file 12906_2023_3960_MOESM10_ESM.pdf]
